# MicroRNA Clusters in the Adult Mouse Heart: Age-Associated Changes

**DOI:** 10.1155/2015/732397

**Published:** 2015-06-28

**Authors:** Xiaomin Zhang, Gohar Azhar, Emmanuel D. Williams, Steven C. Rogers, Jeanne Y. Wei

**Affiliations:** ^1^Reynolds Institute on Aging and Department of Geriatrics, University of Arkansas for Medical Science, 4301 West Markham Street, No. 748, Little Rock, AR 72205, USA; ^2^Geriatric Research Education and Clinical Center, Central Arkansas Veterans Healthcare System, Little Rock, AR 72205, USA

## Abstract

The microRNAs and microRNA clusters have been implicated in normal cardiac development and also disease, including cardiac hypertrophy, cardiomyopathy, heart failure, and arrhythmias. Since a microRNA cluster has from two to dozens of microRNAs, the expression of a microRNA cluster could have a substantial impact on its target genes. In the present study, the configuration and distribution of microRNA clusters in the mouse genome were examined at various inter-microRNA distances. Three important microRNA clusters that are significantly impacted during adult cardiac aging, the miR-17-92, miR-106a-363, and miR-106b-25, were also examined in terms of their genomic location, RNA transcript character, sequence homology, and their relationship with the corresponding microRNA families. Multiple microRNAs derived from the three clusters potentially target various protein components of the cdc42-SRF signaling pathway, which regulates cytoskeleton dynamics associated with cardiac structure and function. The data indicate that aging impacted the expression of both guide and passenger strands of the microRNA clusters; nutrient stress also affected the expression of the three microRNA clusters. The miR-17-92, miR-106a-363, and miR-106b-25 clusters are likely to impact the Cdc42-SRF signaling pathway and thereby affect cardiac morphology and function during pathological conditions and the aging process.

## 1. Introduction

The regulation of cardiomyocyte proliferation, hypertrophy, and function during normal development, maturation, and adult aging is a continuous and progressive process that results in changes in the structure and function of the myocardium and blood vessels, which in turn lead to an increased incidence of cardiovascular diseases with advancing age [1]. It has been documented that the altered expressions of a number of cardiac genes are involved in response to stress and in a number of pathological conditions, as well as in the aging process. These genes include the muscle-specific genes (ANF, *β*-MHC, skeletal *α*-actin, *α*-MHC, cardiac *α*-actin, and SERCA2) [2–5] and transcription factors and cofactors (SRF, myocardin, p49) [6–10], as well as genes regulating metabolism and the extracellular matrix (ECM) [11–13]. Recent studies indicate that noncoding RNAs, especially microRNAs (miRNAs), play an important role in the regulation of messenger RNA (mRNA) expression [14–18].

MicroRNAs (miRNAs) are relatively short (20 to 23 nucleotides), endogenous, and single-stranded RNA molecules that regulate gene expression usually by hybridizing to messenger RNAs (mRNAs) with the consequence of mRNA degradation or translational inhibition of targeted transcripts. Genes that encode for miRNA are transcribed by either RNA polymerase II or RNA polymerase III into primary miRNA (pri-miRNA) transcripts, which are then cleaved by the nuclear microprocessor complex formed by the RNase III enzyme Drosha (RNASEN) and the DGCR8 (DiGeorge critical region 8) protein. The RNase III Dicer cleaves off the loop of the pre-miRNA to generate a roughly 22-nucleotide miRNA duplex [19]. Genes that encode for miRNAs are distributed across chromosomes either individually or in clusters, in which two or more miRNA genes are located within a short distance on the same segment of a chromosome [20, 21]. In the mouse genome, each miRNA cluster contains from two to as many as 71 miRNA genes [22]. The miRNAs within the same cluster are likely to be in the same RNA transcript. In addition, the miRNAs within a single gene cluster may share the same “seed” sequence or may have high sequence homology [21]. Therefore, the increase or decrease in the expression of a miRNA cluster could potentially have a substantial regulatory effect on the posttranscriptional regulation of protein-encoding genes.

In our previous study, we reported altered expression of miRNAs with aging in the adult mouse heart, in which approximately half of the age-related miRNAs were in miRNA clusters, suggesting that these miRNA clusters likely play important roles in the process of cardiac aging [22]. In the present study, we utilized the newly released miRBase database version 21 to examine the miRNA clusters in the mouse genome and observed that 30% of the mouse miRNAs in the current database were clustered miRNAs. We also studied the miRNA clusters that were previously reported to be significantly altered in the old versus young adult mouse heart, especially three related miRNA clusters: the miR-17-92 cluster and its two paralogs, the miR-106a-363 cluster and miR-106b-25 cluster. The expressions of miRNA strands from both the 5′ arm and 3′ arm were considered, and the potential DNA methylation regions were identified. Since the metabolic response to stress may change during the aging process, the effect of nutrient change on miRNA expression was evaluated.

## 2. Materials and Methods

### 2.1. Bioinformatics Analysis

The miRBase (http://www.mirbase.org/) database was used to obtain the 1193 mouse miRNAs from the miRBase database version 21 (June 2014) [23]. The miRBase search tools were used to locate the miRNAs on each chromosome and to search the miRNA clusters with various inter-miRNA distances (1 Kb, 2 Kb, 3 Kb, 5 Kb, and 10 Kb). TargetScan (http://www.targetscan.org/) and http://www.microrna.org/ databases were used to search target mRNAs [24, 25]. The miRNA gene promoter sequence was analyzed using EMBOSS CpGPlot software [26].

### 2.2. Animal Tissues

Healthy C57BL/6 mice were obtained from colonies maintained by the National Institute of Aging (NIA) of the National Institutes of Health, under contractual agreement with Harlan Sprague-Dawley, Inc. (Harlan, IN). After euthanasia, the hearts were removed from mice and subjected to standard RNA isolation and histological procedures. Some heart tissue samples (4 months and 24 months) were obtained from the Aged Rodent Tissue Bank at NIA. For each time point, there were three independent biological replicates. The studies were conducted with the approval of the Institutional Animal Care and Use Committee (IACUC) at Central Arkansas Veterans Healthcare System (IACUC# 4-02-03) and in accordance with the NIH Guiding Principles for Research Involving Animals and Human Beings.

### 2.3. Total RNA Isolation

All RNA samples were first isolated from the mouse cardiac ventricles using UltraSpec RNA Isolation Reagent as previously described. To minimize mouse DNA contamination and enrich the small RNA fraction, the total RNA samples were purified using miRNeasy Mini Kit (Qiagen) and RNase-free DNase I according to the manufacture's instruction manual [10, 27].

### 2.4. miRNA Arrays

The ventricular tissue samples used for the miRNA array analysis were obtained from healthy young adult (4-month-old) and healthy old (24-month-old) C57BL6 mice. The RNA sample isolation and miRNA array were performed in triplicate for young adult and old animals. A total of six RNA samples of ventricular tissue representing three young adult and three old mice were shipped on dry ice to Exiqon, Inc., which provided the service for RNA quality verification, miRNA array hybridization, and comprehensive statistical analysis. The microarray data have been deposited in the NCBI Gene Expression Omnibus (GEO) database (http://www.ncbi.nlm.nih.gov/geo/) under accession number GSE32935. Briefly, each pair of young adult and old mouse samples were labeled with Hy3 and Hy5 fluorescent dyes, respectively, and hybridized to a miRCURY LNA Mouse miRNA Array (version 11.0), which held 648 mature miRNA probes, as well as perfectly matched and mismatched probes for quality control. After signal amplification, the background was subtracted and normalized using LOWESS (locally weighted scatter plot smoothing) regression algorithm. This within-slide normalization was performed to minimize potential differences between the colors in an intensity-dependent manner.

The array output was received in Excel spreadsheets containing the normalized miRNA expression profiles in each heart sample; the expression comparison between old versus young adult heart samples and “expression matrix” containing normalized Hy3/Hy5 ratios (log2 transformed) from all hybridizations was also included. The list was sorted based on the most variant expressed miRNAs comparing the two sample types. 65 miRNAs passed the filtering criteria with an average “log median ratio” >0.58, which represents at least >1.5-fold change in miRNA expression, and the differential miRNA expression in all three pairs (young adult versus old) being in the same direction.

### 2.5. Nutrient Stress Assay

The DMEM media containing 10% newborn bovine serum (Invitrogen) with three different glucose concentrations were used in the present study, the normal glucose (100 mg/dL) medium, the low glucose (30 mg/dL) medium, and the high glucose (400 mg/dL) medium. The muscle cell line C2C12 cells (ATCC CRL-1772) were cultured in six-well plates to 80% confluence in normal glucose medium, after which the cells either remained in normal glucose medium (control) or were subjected to high (400 mg/dL) or low (30 mg/dL) glucose for six hours, respectively. The cells were then harvested and the total RNA was isolated using miRNeasy Mini Kit (Qiagen) and RNase-free DNase I according to the manufacture's instruction manual. Individual experiments were carried out in triplicate, and the results were reported as averages (mean ± SD) from representative experiments [28].

### 2.6. Real-Time RT-PCR Quantitation of Pri-miRNAs and Mature miRNAs

To quantitate the expression of the miRNA primary transcripts and the miRNA mature forms, real-time RT-PCR was performed. To select a proper internal loading control for RT-PCR, we examined the expression of 5S ribosomal RNA (5S RNA) and U6 snRNA in young adult versus old hearts. We found that the expression of 5S RNA remained unchanged in young adult versus old hearts, while U6 snRNA expression changed significantly in young adult versus old hearts. Therefore, 5S RNA was used as an internal loading control.

#### 2.6.1. Detection of Pri-miRNAs

The primers for the detection of pri-miRNAs were designed using PRISM Primer Express 3.0 software (Applied Biosystems) and synthesized at Integrated DNA Technologies Inc. The first-strand cDNA synthesis was carried out using random hexamer primer, and the PCR was performed using the following primers: pri-mir-17 forward 5′-GCTTTGGCTTTTTCCTTTTTG-3′, pri-mir-17 reverse 5′-CCTCACTGCAGTAGATGCACA-3′; pri-mir-20a forward 5′-CGTGGTGTGTGTGATGTGAC-3′, pri-mir-20a reverse 5′-GCTCGTAATGCAGTAGATGGC-3′; pri-mir-25 forward 5′-CAGTGTTGAGAGGCGGAGAC-3′, pri-mir-25 reverse 5′-TCAGACCGAGACAAGTGCAA-3′; pri-mir-27a forward 5′-TTTGATGCCAGTCACAAATCA-3′, pri-mir-27a reverse 5′-AGCCACTGTGAACACGACTTT-3′; pri-mir-92a-1 forward 5′-GGGATTTGTCGCAATGCTGT-3′, pri-mir-92a-1 reverse 5′-GGTCACAATCCCCACCAAAC-3′; pri-mir-93 forward 5′-CACCTCACCTAATGACCCTCA-3′, pri-mir-93 reverse 5′-CAAGTCCTAGCCCTCATGGAT-3′; pri-mir-106a forward 5′-TAAATGCCCCTTCTCGCACA-3′, pri-mir-106a reverse 5′-GGCGAAACACTGAAAGAGCC-3′; pri-mir-106b forward 5′-CTTCCCTCCTACCAGCCCT-3′, pri-mir-106b reverse 5′-GAGCAGCAAGTACCCACAGT-3′; pri-mir-363 forward 5′-TCTGCATCGTAATGGACACCT-3′, pri-mir-363 reverse 5′-TAATGCCACCAATCCCCACC-3′.

#### 2.6.2. Detection of Mature miRNAs

To detect the mature miRNA strand of either miR or miR^*^, the first-strand cDNA synthesis was carried out using a universal reverse primer and the Universal RT miRNA PCR System (Exiqon). The RT-PCR reagents, the primers for mature miRNAs, and the 5S RNA reference primers that were used as endogenous controls were purchased from Exiqon.

The PCR amplification was performed in a 7900HT Fast Sequence Detector System (Applied Biosystems) with the following program: Cycle 1, 95°C for 10 minutes; Cycle 2, 40 cycles of 95°C for 15 seconds, 60°C for 60 seconds; Cycle 3, 95°C for 15 seconds, 60°C for 15 seconds, 95°C for 15 seconds. CT values were automatically obtained. Relative expression values were obtained by normalizing CT values of the miRNA genes in comparison with CT values of the endogenous control (5S RNA) using the CT method [29, 30].

### 2.7. Statistical Analysis

Data are given as mean values ± SD, with *n* denoting the number of experiments unless otherwise indicated. The differentially expressed miRNAs with at least a 1.5-fold change were identified using a *t*-test with a cut-off *P* value of *P* < 0.05.

## 3. Results

### 3.1. An Overview of miRNA and miRNA Clusters in Mouse Genome

**Figure 1:**
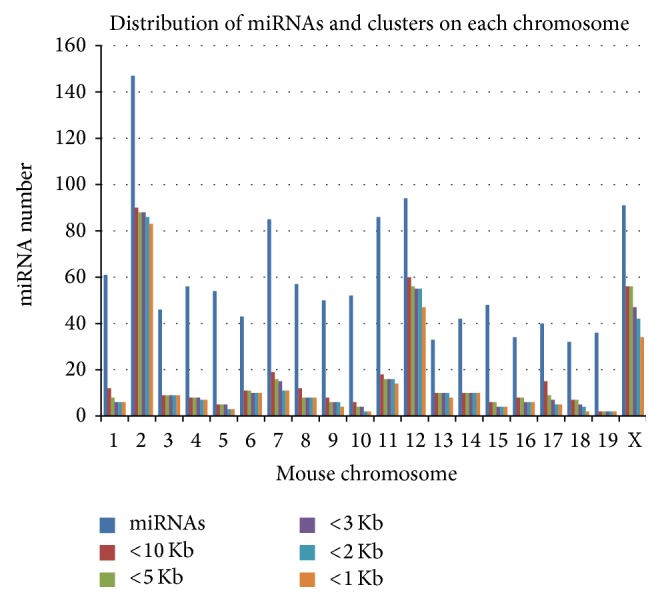
The distribution of mouse microRNAs (miRNAs) and miRNA clusters in each chromosome (chr), based on miRBase version 21. The distribution of miRNA clusters was compared at various inter-miRNA distances (1 Kb, 2 Kb, 3 Kb, 5 Kb, and 10 Kb). Three chromosomes, chr 2, chr 12, and chr X, host most (2/3) of the miRNA clusters, indicating that miRNA clusters are not randomly distributed but rather are enriched at certain genomic locations.

The number of miRNAs that have been deposited in the miRNA database has progressively increased over the past several years. The recently released (June 2014) miRBase database version 21 hosts 1193 mouse miRNA sequences. To examine the potential genomic distribution of the miRNAs in the mouse genome, we utilized the web tool in miRBase (http://www.mirbase.org/) database to search the miRNAs by genomic location and the miRNA clusters based on base pair distance between neighboring miRNAs. As shown in Table 1, 1193 miRNAs were distributed across the mouse chromosomes, with chromosome 2 hosting the highest number of miRNAs. When using 10 Kb as a default distance for clustering neighboring miRNAs, 94 miRNA clusters (containing 372 miRNAs) were found, which account for 31% of the total miRNAs (Table 1). To get an in-depth view of the relationship between miRNA cluster distribution and the distance of neighboring miRNAs, we also used distances of 5 Kb, 3 Kb, 2 Kb, and 1 Kb to explore the miRNA clusters. As shown in Table 1 and Figure 1, more than 300 miRNAs (approximately 1/4 of mouse miRNAs) were distributed in clusters within a 2 Kb distance. Compared to the previous miRBase version 20, the current version 21 had a 39% increase of miRNAs in the mouse genome (Table 2).

### 3.2. The Genomic Loci of miR-17-92 and Its Paralogs and Their Gene Families

In our previous study, we observed that 11 miRNA clusters were significantly impacted in the cardiac aging process; among them were three clusters that belong to miR-17-92 cluster and its paralogs. They are miR-17-92, miR-106a-363, and miR-106b-25 clusters [22]. These data indicate that miR-17-92 and its paralogs may play an important role in the regulation of physiological and structural changes in the adult heart during aging. Due to the similarity of gene sequences of these miRNA clusters, it is speculated that the paralogs, clusters miR-106b-25 and miR-106a-363, are likely to have been derived from the gene duplication of the miR-17-92 cluster [31].

**Figure 2:**
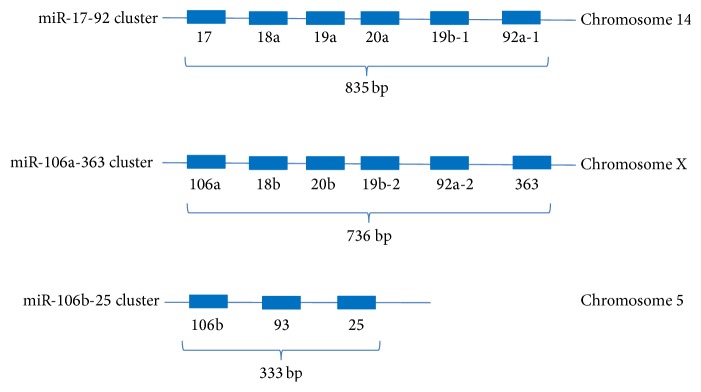
Genomic loci of miR-17-92, miR-106a, and miR-106b-25 clusters. The miR-17-92 cluster spans approximately 825 bp on chromosome 14. The miR-106a-363 cluster spans approximately 736 bp on chromosome X. The miR-106b-25 cluster spans 333 bp on chromosome 5.

In the mouse genome, the miR-17-92 cluster is located on chromosome 14, the miR-106a-363 cluster is on chromosome X, and miR-106b-25 cluster is on chromosome 5 (Figure 2). The gene loci of all 3 clusters span less than 1 Kb, indicating that the miRNAs in each cluster are likely to be transcribed within one transcript.

The entire mouse miR-17-92 cluster is transcribed within a RNA transcript of 2339 bp in length, termed “Mir17 host gene 1” (GenBank accession number NR_029382), which is the only documented transcript variant/isoform. In contrast, the human miR-17-92 cluster has two reported host gene variants/isoforms: (1) the “miR-17 host gene 1,” which is 5018 bp in length (NR_027350) and contains all 6 miRNAs of the miR-17-92 cluster, and (2) the “miR-17-92 cluster host gene variant 2,” which is 927 bp in length (NR_027349) and apparently does not have perfect sequence alignment with any of the miRNAs in the cluster.

The three miR-17-92-related clusters hosted 15 miRNA genes (Figure 2) and produced 15 mature miRNAs (guide strand) and 15 passenger strands (Table 3). These miRNAs were categorized into four miRNA families in the miRBase database, based on their sequence similarity of the “seed” sequence. As shown in Table 4, eight miRNAs were observed to belong to the miR-17 family; three miRNAs were identified to belong to the miR-19 family and three miRNAs to the miR-25 family; the miR-363-5p was found to belong to the miR-363 family, but its sequence was similar in part to that of miR-25 family members (Table 3).

### 3.3. The Expression of miR-17-92 and Its Paralogs in the Old Heart

**Figure 3:**
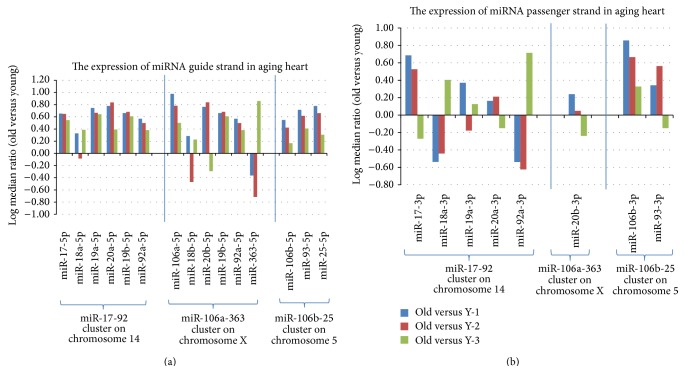
Expression of miR-17-92, miR-106a-363, and miR-106b-25 clusters in old versus young adult mouse hearts. (a) The expression of miRNA guide strands with aging in the heart. Most of the 15 guide strands were upregulated with aging in the heart, where 11 guide strands were expressed in the same direction, but four guide strands (miR-18a-5p, miR-18b-5p, miR-20b-5p, and miR-363-5p) were increased in some old mice while decreased in other old mice. (b) The expression of miRNA passenger strands with aging in the heart. The expression of the eight passenger strands was complex, where only one, miR-106a-3p, was increased in all three old versus young adult mice, while seven passenger strands were increased in some old mice and decreased in other old mice.

The miR-17-92 cluster and its paralogs are usually first transcribed as primary transcripts (pri-miRNA) which contain the miRNA genes. The pri-miRNAs are cleaved by the microprocessor complex into pre-miRNA, which is a short stem loop ~70 nucleotides in length. The pre-miRNA is then cleaved by the RNase III enzyme Dicer and becomes mature miRNAs as a guide strand (miR) and a passenger strand (miR^*^) [32]. During typical miRNA biogenesis, the miR strand is preferentially selected for entry into a RISC complex, whereas the miR^*^ strand has been thought to be degraded [33–35]. However, growing evidence now indicates that the miRNA passenger strand may also have important biological functions [36]. Therefore, the expression of both the guide and passenger strands was examined in the hearts of old versus young adult mice. Exiqon miRNA chip covered all the 15 guide strands from the three miRNA gene clusters (Figure 3(a)) and covered more than half of the passenger strands (Figure 3(b)). Most of the 15 guide strands were upregulated in the aging heart, where 11 guide strands were expressed in the same direction, but four guide strands (miR-18a-5p, miR-18b-5p, miR-20b-5p, and miR-363-5p) were increased in some old mice while decreased in other old mice (Figure 3(a)). The expression of the eight passenger strands was more complex, where only one, miR-106a-3p, was increased in all three old versus young adult mice, while seven passenger strands were increased in some old mice and decreased in other old mice (Figure 3(b)).

### 3.4. miRNA Expression in Response to Stress

**Figure 4:**
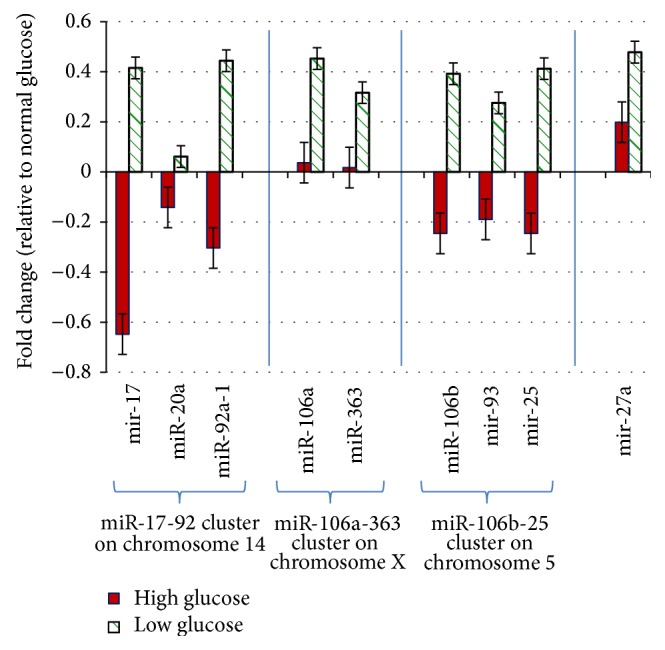
The miRNA expression in response to glucose stress. The C2C12 cells (ATCC CRL-1772) were cultured in normal glucose (100 mg/dL) medium to 80% confluence and then treated with either high glucose (400 mg/dL) or low glucose (30 mg/dL) for six hours, respectively. The miR-17-92 cluster is represented by three microRNAs; the miR-106a-363 cluster is represented by two microRNAs; the miR-106b-25 cluster is represented by three microRNAs. The mircroRNAs in each of the clusters either increased or decreased in the same direction in response to the stresses. Please note that miR-27a does not belong to any of the miR-17-92-related clusters but has been included here for comparison only.

The metabolic response to stress tends to change during adult aging. To examine whether nutrient stress could have an effect on miRNA expression, miRNA expression was measured in cultured cells in response to high and low glucose versus normal glucose (control) treatment. The expression of two or three microRNAs was measured in each of the miR-17-92-related clusters. As shown in Figure 4, after six hours of high glucose (400 mg/dL) and low glucose (30 mg/dL) treatment, the miR-17, miR-20a, and miR-92a-1 from miR-17-92 cluster were decreased in response to high glucose but increased in response to low glucose. The miR-106a and miR-363 from miR-106a-363 cluster were increased in response to both high and low glucose stresses. The miR-106b, miR-93, and miR-25 from miR-106b-25 cluster were decreased in response to high glucose while increased in response to low glucose treatment. The miR-27, which does not belong to any of the miR-17-92-related clusters, was increased in response to both high and low glucose stresses.

### 3.5. Analysis of the miRNA Gene Promoter and miRNA Target Genes

**Figure 5:**
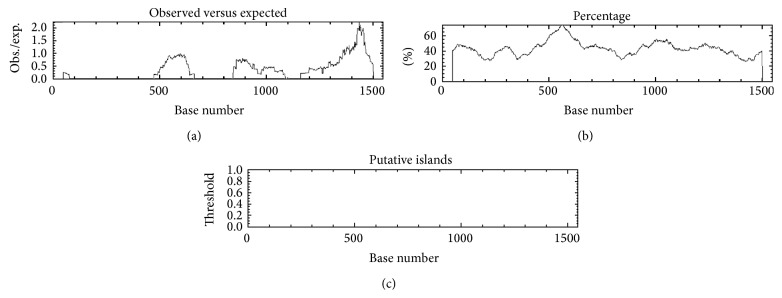
Analysis of the miR-17-92 cluster promoter region using “cpgplot” web tool (http://www.ebi.ac.uk/Tools/emboss/cpgplot/) to search the CpG island/GC-rich region, which revealed the GC-rich region in the promoter region.

It has been reported that miRNA gene promoters are frequent targets of DNA methylation under various physiological and pathological conditions [37–39]. Since altered DNA methylation has been reported in cardiac disease and during the adult aging process, the GC-rich region in the promoter of miR-17-92 cluster was further examined. As shown in Figure 5, the miR-17-92 promoter has a high GC-rich region, suggesting that the miR-17-92 cluster could quite possibly also be regulated by DNA methylation.

**Figure 6:**
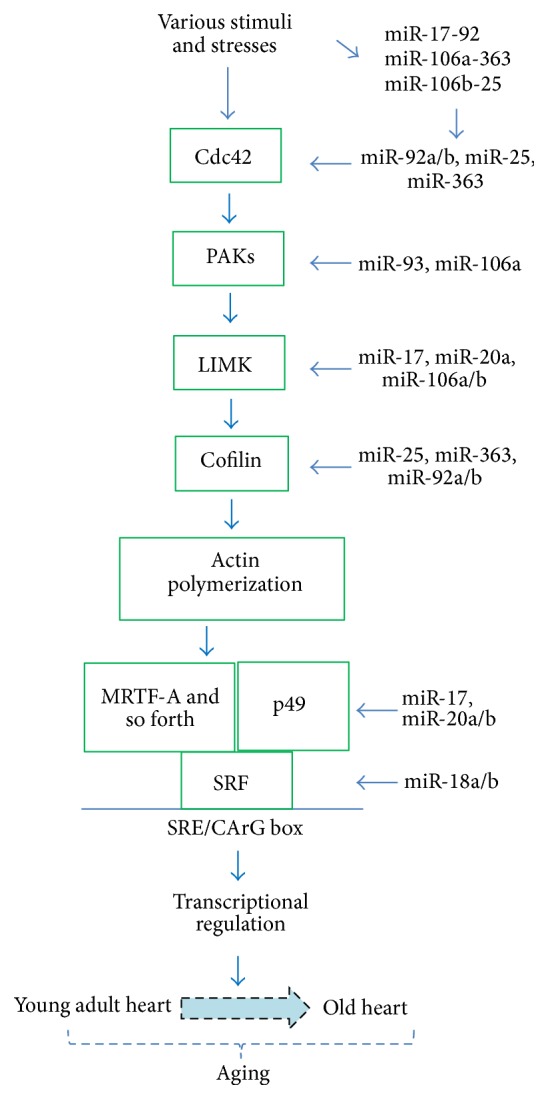
Cdc42-SRF signaling pathway regulates cytoskeletal genes that are important in the development and maintenance of cardiovascular structure and function. The schematic here is a simplified presentation of the cdc42 pathway with some of the main proteins, which are potential targets of the miRNAs from the miR-17-92 cluster and its paralogs, the miR-106a-363 and miR-106b-25 clusters. The targeting information has been obtained from the literature and from the bioinformatics search with TargetScan and http://www.microrna.org/ databases.

The Cdc42-SRF signaling pathway is important for the development and maintenance of the cardiovascular system. Potential signaling proteins that are likely targeted by the miR-17-92 cluster and its paralogs were analyzed using TargetScan and http://www.microrna.org/ web tools. The schematic diagram shown in Figure 6 is a simplified presentation of the Cdc42-SRF pathway that has been reported to be targeted by miRNAs based on the present study and other published reports in the literature.

## 4. Discussion

In the present study, the miRNA clusters in the mouse genome were examined based on their chromosomal location and various inter-miRNA distances. Three important miRNA clusters that were identified to be significantly impacted during the cardiac aging process, the miR-17-92 cluster and its paralogs, miR-106a-363 and miR-106b-25, were also examined in terms of their genomic location, RNA transcript character, sequence homology, and their relationship with the corresponding miRNA families. The expression of both the guide and passenger strands of the miRNAs was evaluated in the old compared to that of young adult mouse hearts. Since response to various stresses is implicated in the process of aging and in the development of disease in the heart, the effect of glucose stresses on the expression of miRNA clusters was also evaluated. In addition, a high GC-rich region in the promoter region of miR-17-92 cluster was identified, which indicates that DNA methylation could also be a potential mechanism of regulation of miRNA cluster expression during aging in the heart. The data indicate that miR17-92 cluster and its paralogs, miR-106a-363 and miR-106b-25, potentially target the cd242-SRF signaling pathway, thereby regulating the cardiac response to pathophysiological conditions, including hypertension, cardiac hypertrophy, heart failure, and arrhythmias.

The number of miRNAs collected in miRNA databases has been growing steadily over the past several years, with both increased number of miRNA entries and increased coverage of species. For instance, miRBase released its miRNA database version 1.0 in December 2002, which had only 218 entries. However, the version 20 had 24,521 entries, while the most recently released (June 2014) version 21 has increased to up to 28,645 entries. Similar to many other species, the miRNAs listed in the mouse genome have also increased over time. There were 855 mouse miRNAs reported in version 20, but this number has significantly increased to 1193 miRNAs in version 21. Similarly, the number of miRNA clusters has also increased over time (Table 2).

The inter-miRNA distance used for grouping miRNA clusters has varied among different reports, with ranges from 1 Kb to 50 Kb [21, 22, 40]. However, most miRNA clusters are located within 1–3 Kb inter-miRNA distance [21, 41]. In the present study, we observed that more than 20–25% of the miRNAs were within 1-2 Kb distance from each other, and more than 30% of the miRNAs were within a 10 Kb distance from each other. Since the length of the RNA transcript typically varies from 1 Kb to over 10 Kb, many of these miRNA clusters may have one core promoter region and the same transcriptional start site and are perhaps expressed within a single RNA transcript [42]. We also observed that the miRNAs and miRNA clusters are not evenly distributed across the chromosomes. For instance, three chromosomes, chromosome 2, chromosome 12, and chromosome X, hosted over 2/3 of the miRNA clusters, suggesting that the distribution of miRNA clusters is not random but rather is enriched at certain genomic locations.

The miRNAs have been implicated in the processes of cardiac hypertrophy and heart failure [43–48]. Left ventricular hypertrophy and heart failure are known to increase significantly in prevalence with advancing age. The older heart is characterized by a progressive loss of myocytes with subsequent hypertrophy of the remaining cells and increased fibrosis with collagen deposition, as well as calcification involving the conduction and valvular apparatus [1, 49, 50]. The old heart is more vulnerable to environmental changes, including dynamic stress, hypoxia, ischemia-reperfusion, hyperglycemia, and/or hypoglycemia.

Multiple signaling pathways are likely involved in the lifelong process of cardiac aging, one of which is the Rho-SRF signaling pathway [51–53]. Serum response factor (SRF) has been shown to be a major transcription factor in the regulation of the genes involved in the maintenance of cardiac structure and function [54–57]. Cardiac-specific overexpression of SRF at a mild level caused an accelerated cardiac aging phenotype in mice [9]. A number of other mouse models also highlight the importance of SRF and its related signaling pathway in the regulation of cardiac structure and function during embryogenesis, maturation, and aging [8, 9, 56–59].

SRF is a downstream effector of the Rho family GTPases [60–62]. The Rho GTPases (Cdc42, Rac1, and RhoA) signaling pathways regulate cytoskeletal genes that are important in the development and maintenance of cardiovascular structure and function as well as the processes of pathological conditions [63, 64]. The miRNAs in the three miR-17-92-related clusters apparently target multiple components of the Rho GTPase-related signaling pathways, including the Cdc42-SRF signaling pathway (Figure 6) [65].

Cdc42 is an upstream regulator in the pathway, which has been implicated in regulating myofibrillar architecture, cell polarity, and morphology, as well as sarcomere assembly in the cardiac myocyte [64, 66–68]. Cdc42 is a target of miR-92a and miR-92b and miR-25 and miR-363. The p21 protein (Cdc42/Rac) activated kinases (PAKs) are targets of Cdc42/Rac and play a critical role in proper morphogenesis, including normal electrical conductance of the heart, cardiac contractility, and development and maintaining of the integrity of the vasculature [65]. PAK2 is a target of miR-93 and miR-106a; PAK6 is a target of miR-19a and miR-19b [69]. LIM-kinases (LIMKs) are serine/threonine-protein kinases that play an essential role in the regulation of actin filament dynamics by phosphorylating cofilins. Cofilins are actin binding/depolymerizing factors which can polymerize and depolymerize F-actin and G-actin [70, 71]. LIM-kinase 1 (LIMK1) is a target of miR-20a and other miRNAs including miR-106a, miR-106b, miR-17, miR-20a, miR-20b, and miR-93 [72]. Cofilin 2 is a target of miR-25, miR-363, miR-92a, and miR-92b. The p49/STRAP (SRFBP1) gene is a target of miR-106a, miR-106b, miR-17, miR-20a, miR-20b, and miR-93. The SRF gene is a target of miR-18a and miR-18b. Therefore, altered expression of the miR-17-92, miR-106a-363, and miR-106b-25 clusters is likely to sufficiently and significantly impact the Cdc42-SRF signaling pathway and thereby affect cardiac structure and function during pathological conditions and the aging process.

The heart comprises cardiomyocytes, fibroblasts, endothelial cells, and vascular smooth muscle cells. The cardiomyocytes account for 30–35% of cell population, while noncardiomyocytes account for 65–70% of the cell population of the heart in human and rat [73–75]. Due to the species difference, however, the young adult murine myocardium is composed of 56% myocytes, 27% fibroblasts, 7% endothelial cells, and 10% vascular smooth muscle cells [76]. The cardiac fibroblasts tend to increase to cause cardiac fibrosis and induce collagen deposition in the aging heart as well as in the hypertrophied heart. Cardiac fibroblasts can also secrete miRNA-enriched exosomes which contain a relatively high abundance of many miRNA passenger strands, including miR-21^*^. The miR-21^*^ has been shown to induce cardiomyocyte hypertrophy [36]. Therefore, the microRNAs may serve as paracrine signaling mediators of cellular responses, including cardiomyocyte hypertrophy. The analysis of microRNAs from each of the cell populations of the heart in response to cardiac stress would be of interest, as would the consideration of promoter regions of the microRNA cluster in terms of GC-rich regions. These questions warrant future investigation.

## 5. Conclusions

It is becoming increasingly clear that the miRNAs and miRNA clusters are important in regulating an animal's response to environmental stimuli, including nutrient stress, the maintenance of normal function, and the development of pathological conditions, as well as the process of aging. Inasmuch as Cdc42-SRF signaling pathway, including serum response factor (SRF) and its cofactors, has been observed to mediate the cellular responses to a variety of external and internal stimuli, those miRNAs that modulate and/or are modulated by components of the Rho GTPase-related signaling pathways are likely to be attractive targets for future therapeutic considerations.

## Figures and Tables

**Table 1:** The number of mouse miRNAs and miRNA clusters distributed on each chromosome. A total of 1193 miRNAs were observed in the mouse miRBase database release version 21. Six miRNAs were not assigned to any of the chromosomes. Various inter-miRNA distances (1 Kb, 2 Kb, 3 Kb, 5 Kb, and 10 Kb) were used in an effort to define the potential miRNA clusters. Approximately 25% of the miRNA clusters are located within a 2 Kb distance.

Chromosome	Total miRs	Cluster number				
		<10 Kb	<5 Kb	<3 Kb	<2 Kb	<1 Kb
1	61	12	8	6	6	6

2	147	90	88	88	86	83

3	46	9	9	9	9	9

4	56	8	8	8	7	7

5	54	5	5	5	3	3

6	43	11	11	10	10	10

7	85	19	16	15	11	11

8	57	12	8	8	8	8

9	50	8	6	6	6	4

10	52	6	4	4	2	2

11	86	18	16	16	16	14

12	94	60	56	55	55	47

13	33	10	10	10	10	8

14	42	10	10	10	10	10

15	48	6	6	4	4	4

16	34	8	8	6	6	6

17	40	15	9	7	5	5

18	32	7	7	5	4	2

19	36	2	2	2	2	2

*X*	91	56	56	47	42	34

Other^*^	6					

Cluster number		**94**	**87**	**84**	**81**	**93**

Total miRs	1193	372	343	321	302	275

Cluster %		**31%**	**29%**	**27%**	**25%**	**23%**

^*∗*^Six miRNAs have not been localized into any of the above chromosome.

**Table 2:** The increased identification of miRNAs and miRNA clusters in the mouse genome, according to miRBase database version 20 versus version 21.

miRBase	Release 20	Release 21	Increase
Total miRs	855	1193	39%
Total clusters	71	94	32%
Seq. in clusters	318	372	17%

**Table 3:** The “seed” sequence similarity and miRNA families of miR-17-92 and paralogs. The “seed” sequences of the 15 miRNAs (underlined) are derived from miR-17-92, miR-106a-363, and miR-106b-25 clusters. The mature miRNAs are grouped into four miRNA families, based on their “seed” sequence similarity (miRBase version 21).

I D	5′ arm strand	ID	3′ arm strand
m iR-17 family			
mmu-miR-17-5p	$C\underline{AAAGUGCU}UACAGUGCAGGUAG$	m mu-miR-17-3p	$\underline{ACUGC}AGUGAGGGCACUUGUAG$
mmu-miR-20a-5p	$U\underline{AAAGUGCU}UAUAGUGCAGGUAG$	mmu-miR-20a-3p	$\underline{ACUGC}AUUACGAGCACUUAAAG$
mmu-miR-93-5p	$C\underline{AAAGUGCU}GUUCGUGCAGGUAG$	mmu-miR-93-3p	$\underline{ACUGC}UGAGCUAGCACUUCCCG$
mmu-miR-106b-5p	$U\underline{AAAGUGCU}GACAGUGCAGAU$	mmu-miR-106b-3p	$CCGC\underline{ACUG}UGGGUACUUGCUGC$
mmu-miR-20b-5p	$C\underline{AAAGUGCU}CAUAGUGCAGGUAG$	mmu-miR-20b-3p	$\underline{ACUGC}AGUGUGAGCACUUCUAG$
mmu-miR-106a-5p	$C\underline{AAAGUGCU}AACAGUGCAGGUAG$	mmu-miR-106a-3p	$\underline{ACUGC}AGUGCCAGCACUUCUUAC$
mmu-miR-18a-5p	$U\underline{AAGGUGCAU}CUAGUGCAGAUAG$	mmu-miR-18a-3p	$\underline{ACUGC}CCUAAGUGCUCCUUCUG$
mmu-miR-18b-5p	$U\underline{AAGGUGCAU}CUAGUGCUGUUAG$	mmu-miR-18b-3p	$U\underline{ACUGC}CCUAAAUGCCCCUUCU$
miR-19 family			
mmu-miR-19a-5p	$U\underline{AGUUUUGCA}UAGUUGCACUAC$	mmu-miR-19a-3p	$\underline{UGUGCAAAUC}UAUGCAAAACUGA$
mmu-miR-19b-1-5p	$\underline{AGUUUUGCA}GGUUUGCAUCCAGC$	mmu-miR-19b-3p	$\underline{UGUGCAAAUC}CAUGCAAAACUGA$
mmu - miR-19b-2-5p	$\underline{AGUUUUGCA}GAUUUGCAGUUCAGC$	mmu-miR-19b-3p	$\underline{UGUGCAAAUC}CAUGCAAAACUGA$
miR-25 family			
mmu-miR-25-5p	$\underline{AGG}CGGAGACUUGGGCAAUUGC$	mmu-miR-25-3p	$C\underline{AUUGCACU}UGUCUCGGUCUGA$
mmu-miR-92a-1-5p	$\underline{AGG}U\underline{UGG}GAUUUGUCGCAAUGCU$	mmu-miR-92a-3p	$U\underline{AUUGCACU}UGUCCCGGCCUG$
mmu-miR-92a-2-5p	$\underline{AGGUGG}GGAUUGGUGGCAUUAC$	mmu-miR-92a-3p	$U\underline{AUUGCACU}UGUCCCGGCCUG$
mir-363 family			
mmu-miR-363-5p	$C\underline{AGGUGG}AACACGAUGCAAUUU$	mmu-miR-363-3p	$A\underline{AUUGCAC}GGUAUCCAUCUGUA$

**Table 4:** The miRNA families and locations of the miR-17-92 cluster and paralogs. The miRNA families of the miR-17-92 cluster and its paralogs. The miRNAs in the miR-17-92 cluster and its paralogs are grouped into four families based on the data in the miRBase database. The mouse miR-17 family has eight members, which are from three clusters. The mouse miR-19 family has three members, which are from two clusters. Three members of the miR-17-92 cluster and its paralogs belong to the miR-25 family. The miR-363 family currently has only one mouse miRNA member.

miR ID	Cluster	Chromosome	Accession
mir-17 family			
mmu-mir-17	miR-17-92 cluster	chr14	MI0000687
mmu-mir-18a	miR-17-92 cluster	chr14	MI0000567
mmu-mir-18b	miR-106a-363 cluster	chrX	MI0005483
mmu-mir-20a	miR-17-92 cluster	chr14	MI0000568
mmu-mir-20b	miR-106a-363 cluster	chrX	MI0003536
mmu-mir-93	miR-106b-25 cluster	chr5	MI0000581
mmu-mir-106a	miR-106a-363 cluster	chrX	MI0000406
mmu-mir-106b	miR-106b-25 cluster	chr5	MI0000407
miR-19 family			
mmu-mir-19a	miR-17-92 cluster	chr14	MI0000688
mmu-mir-19b-1	miR-17-92 cluster	chr14	MI0000718
mmu-mir-19b-2	miR-106a-363 cluster	chrX	MI0000546
miR-25 family			
mmu-mir-25	miR-106b-25 cluster	chr5	MI0000689
mmu-mir-92a-1	miR-17-92 cluster	chr14	MI0000719
mmu-mir-92a-2	miR-106a-363 cluster	chrX	MI0000580
mir-363 family			
mmu-mir-363	miR-106a-363 cluster	chrX	MI0000765
